# Getting ahead of the pandemic curve: A systematic review of critical determining factors for innovation adoption in ensuring food security

**DOI:** 10.3389/fnut.2022.986324

**Published:** 2022-11-03

**Authors:** Ammar Redza Ahmad Rizal, Shahrina Md Nordin

**Affiliations:** ^1^Faculty of Social Sciences and Humanities, Centre for Research in Media and Communication, National University of Malaysia, Bangi, Malaysia; ^2^Institute Self-Sustainable Building, University of Technology PETRONAS, Seri Iskandar, Perak Darul Ridzuan, Malaysia

**Keywords:** food crop, innovation, adoption, Preferred Reporting Items for Systematic Reviews and Meta-Analyses (PRISMA), food security, diffusion, farmers

## Abstract

The imminent threat to food security requires immediate intervention toward ensuring societal sustainability especially in combating the pandemic. The rapid spread of COVID-19 cases has caused concern for food security. A recent outlook report produced by Food Agricultural Organization and World Food Programme (FAO-WTP) highlights that there are at least 20 countries that are faced with a looming threat of food availability between the period of March-July 2021. Other factors that pose a significant threat to food security include climate change and natural disasters which could significantly reduce the yield. It is hence imperative to gain an in-depth understanding of factors that influence farmers’ choices in innovation adoption for increased yield. A line of research has been conducted across the globe on new technology adoption and effect of innovation that aims to increase productivity and yield. This study examined the key factors, that lead farmers to the adoption of new technology and innovation, reported in studies over the past 15 years. PRISMA-P (Preferred Reporting Items for Systematic Review and Meta-Analysis Protocols) was employed based on the SCOPUS and Web of Science database. In creating the main dataset, a protocol was developed in advance to document the analysis method. Several inclusion (eligibility) and exclusion criteria were set to select related articles from a total of 2,136 papers. The thematic and content analyses were subsequently performed on 392 research articles. The findings indicate 4 over-arching segments, and 12 major determinants, that comprise 62 associate determinants. The paper concludes with the identification of critical factors for innovation adoption amongst farmers.

## Introduction

The imminent threat to food security requires immediate intervention toward ensuring societal sustainability. A recent outlook report produced by Food Agricultural Organization and World Food Programme (FAO-WTP) highlights that there are at least 20 countries that are faced with a looming threat of food availability between the period of March–July 2021 (1). FAO further reported that 45 countries are in need of external assistance for food mostly due to the COVID-19 pandemic, which has severely aggravated global food security conditions (2). Despite the arguments that global calorie intake has shifted toward a more diversified diet that includes higher shares of meat, dairy products, fats, sugar, fruit and vegetables (3), staple food crops are still in demand. It is also reported that 50% of daily calorie intake is derived directly from cereal grain and staple food crops consumption (4).

Cereal grain and staple food crops are not only essential for human physiological demand, but they also act as a core economic driver for both society and a country. It is estimated that the annual global trade for cereal grain is pegged at 441 million tonnes (2), approximately 200 billion USD in terms of the crop trade value alone (see Table 1 for the details). There is however, a serious threat of scarcity in staple food supply across many countries that are caused by numerous factors including climate change-related issues instance as water scarcity and natural disaster (5); COVID-19 pandemic (6); and rapid urbanization (7).

**TABLE 1:** Basic facts of world cereal grain.

Million tonnes	2018/19	2019/20 estimate	2020/21 forecast	Change: 2020/21 over 2019/20 (%)
*Production*	2,645.9	2,706.3	2,764.9	2.2
Developing countries	1,614.0	1,648.8	1,678.6	1.8
Developed countries	1,032.0	1,057.5	1,086.3	2.7
*Trade*	410.4	434.3	441.4	1.6
Developing countries	144.3	163.5	160.6	-1.8
Developed countries	266.1	270.8	280.8	3.7
*Utilization*	2,674.9	2,683.3	2,746.4	2.4
Developing countries	1,814.0	1,827.8	1,874.3	2.5
Developed countries	860.9	855.5	872.2	1.9
Per capita cereal food use (kg per year)	149.6	149.7	150.1	0.3
*Stocks*	868.1	880.9	895.5	1.7
Developing countries	677	691.5	696.7	0.8
Developed countries	191.2	189.4	198.8	4.9
World stock-to-use ratio (%)	32.4	32.1	31.8	-0.8

Data obtained from Food Agriculture Organization (2) Report – “Crop Prospects and Food Situation: Quarterly Report”.

The gap between staple crop production and the demand for human food consumption has widened over the decades (3). There is hence a dire need to increase production that could be made possible through innovations. Green Revolution was previously implemented by several countries such as China, India, Malaysia, and other developing countries in the 1960s and 1970s. In the recent development of innovation in staple food crop farming, attention has expanded to include areas such as green technology, sustainable farming, and conservative agriculture.

There has been a line of studies on the diffusion of innovation and adoption of new technology amongst farmers. For instance, Nordin and colleagues identified that agriculture education is a significant determinant for farmers’ agriculture adoption (8). They also found that social media affordances help farmers to reduce complexity in adopting new innovations (9). There are also several other studies that test various perspectives in explaining farmers’ adoption of new innovations including adopting theories of planned behavior (10), identifying factors other than utility theories (11), government support as determining factors (12) and the usage of intention modeling (13). However, efforts to systematically review these studies are still lacking.

Most of the current systematic reviews found focus on the adaptation of efforts related to climate-resilient crops (14), climate change adaptation (5), climate change policy (15), non-agriculture community (16), and focused on other regions (17). This paper seeks to fill the gap in understanding and identifying the characteristics of innovation as well as the determinants of adopting them among staple food crop farmers. Studies, articles, and reports on adoption and diffusion of innovation in the peer-reviewed literature within the database are used in this study.

The review was guided by the main question of what determinants affect staple food crops farmers around the world to adapt to new technology and innovation in lieu of the recent pandemic crisis? The review primarily focuses factors affecting on farmers’ adoption. Focusing on this aspect is pivotal as the world is currently expected to face several other crises including energy and economic crisis. By understanding this matter, it will help policymakers, innovators and stakeholders develop better strategic approaches to increase farmers adoption to innovation.

### Methodology

The method used in this study referred to as PRISMA (18). The review is conducted on peer-reviewed articles found in two of the largest academic databases – SCOPUS and Web of Science (WOS). The approaches to conduct systematic review include identifying eligible and exclusion criteria, steps of the review process (identification, screening, eligibility) and data abstraction analysis.

## Preferred Reporting Items for Systematic Reviews and Meta-Analyses

Preferred Reporting Items for Systematic Reviews and Meta-Analyses (PRISMA) was used as a guide for the review due to its three unique advantages. First, it enables the study to define clear research questions that permit systematic research. The PRISMA method has been used extensively for systematic review studies in social science (18–22). Second, the guidelines enable the identification of inclusion and exclusion criteria. Finally, it supports examining a large database in the scientific literature in a defined time. The PRISMA statement allows for a rigorous search of the term related to staple food crops farmers’ adoption to innovation.

### Resources

The systematic review was based on two main academic databases – SCOPUS and Web of Science (WOS). Both of the databases consist of more than 33,000 journals across 256 disciplines which include disciplines and subjects related to agronomy, multi-disciplinary agriculture, interdisciplinary social sciences, food technology, social issues as well as development and planning. It includes comprehensive research data and citations, established by Clarivate Analytics and ranks them by three separate measures: citations, papers, and citation per paper. The second database is SCOPUS, a database product owned by Elsevier. It has more than 22,800 journals from around 5,000 publishers worldwide (23). Similar to WOS, the SCOPUS index consists of diverse subject areas which is suitable for this systematic review.

### Search protocol – Eligibility and exclusion criteria

To create the main dataset, a protocol was developed in advance to document the analysis method. Several inclusion (eligibility) and exclusion criteria were determined. The first criteria are the literature type, only journal articles with primary data are selected. This means review articles, panel series data, book series, books, and conference proceedings are all excluded. The selection of only journal articles is to ensure only recent findings associated with innovation adoption can be captured in this systematic review. Furthermore, journal articles that are indexed by both databases have been through a rigorous peer review process. This shall ensure the methods used in their study have been validated and thus provide more concrete findings in this systematic review.

Second, to avoid any confusion and loss of meaning in translation, the protocols exclude non-English publication. Thirdly, concerning the timeline, which is sensitive to innovation, a period of 15 years was selected (between 2006 and 2021). This allows recent issues related to innovation adoption to be identified. The next criteria for the search protocol are the areas of research, only research related to staple food crops is selected. Staple food crops in this study include cereal grains (e.g., paddy, wheat, barley, maize, millet, and sorghum) as well as some tuber roots (e.g., yam, potato, and cassava) and plantain. Legumes, beans, and non-food crops are excluded. Finally, the search protocols only focus on adaption by farmers. Table 2 summarizes the inclusion and exclusion criteria.

**TABLE 2:** Protocol inclusion and exclusion criteria.

Criterion	Eligibility	Exclusion
Literature type	Journal/Book chapter	Book series, books, conference proceeding
Language	English	Non-English
Timeline	Between 2006 and 2021	<2006
Type of data	Primary data	Secondary data, panel series, systematic review
Crop type	Cereal grain, cassava, yam, potato, plantain	Legumes and beans, non-food crop
Unit of analysis	Farmers	Non-farmers

### Systemic review process

Based on the guideline in PRISMA, four stages were involved in the systematic review process. The search was performed on 2 March 2021. The first phase identified the keywords used in the search process. Based on terminologies used in the past studies, keywords similar and related to innovation adoption, staple food crops and farming community were used. The search string used for the systematic review process is included as supporting materials in this paper.

**FIGURE 1:**
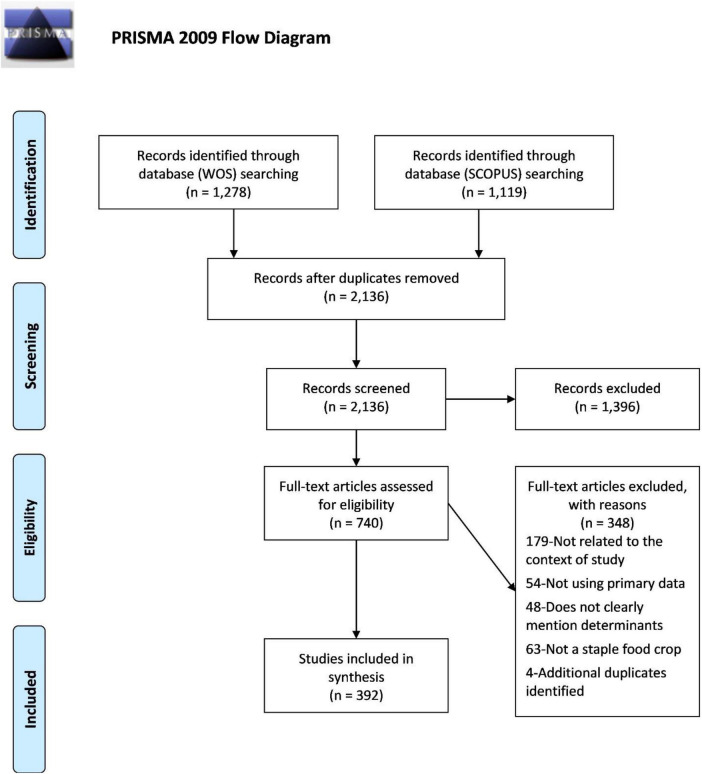
PRISMA flow chart. From Moher et al. (24).

The second stage involved screening. At this stage, out of 2,136 articles eligible to be reviewed, a total of 1,396 articles were removed. The third stage examined eligibility, where the full articles were accessed. After careful examination, a total of 348 articles were excluded due to several factors such as not related to the field of study, not a food staple crop, and not discussing adoption factors. The last stage of review resulted in a total of 392 articles that were used for the qualitative analysis. The screening flow is shown in Figure 1.

### Data abstraction and analysis

The final 392 articles were obtained for rigorous analysis and assessment. The analysis focused on the types of crops and innovations adopted by farmers. Then, the determinants for adoption were identified. The data were extracted by analysing the abstracts, prior to examining the full articles (in-depth), which is essential to identify major determinants and the associate determinants. The qualitative analysis was conducted through content analysis and thematic analysis. It allows categorization of themes and associated sub-themes. The findings will be discussed in the following section.

## Results

**FIGURE 2:**
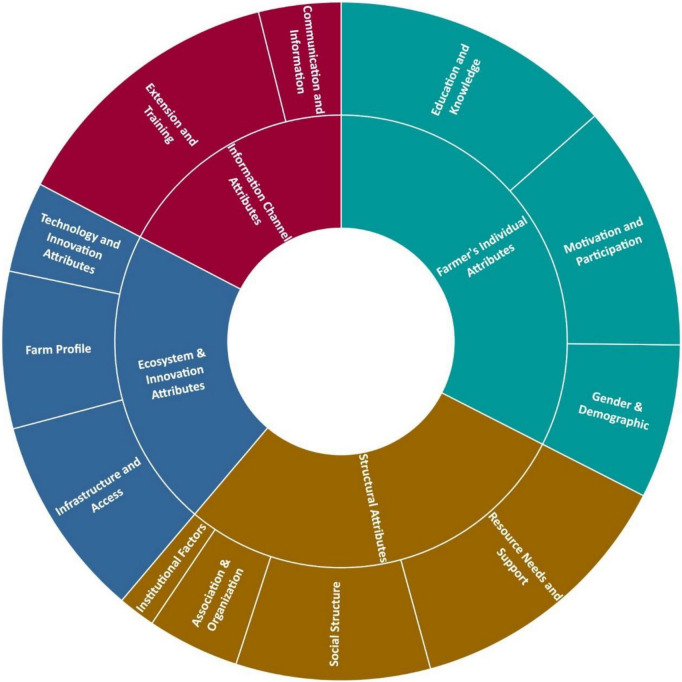
Summary of determinants of new technology adoption by staple food crop farmers.

The review identified the determinants of farmers’ decision-making in adopting new technology or innovation in the production of staple food. Generally, a line of studies reported multiple determinants that lead to farmers’ adoption of new technology. The review resulted in 4 segments comprising of 12 main determinants and 62 associated determinants (see Figure 2). Three main determinants were identified in the first segment – the farmers’ attributes. The determinants are – (1) Education and knowledge; (2) Motivation and participation; (3) Gender and demographics. The second segment is information channel attributes where there are 2 main determinants identified – (1) Extension and training; (2) Communication and information. There are 3 main determinants in the ecosystem and innovation attributes segment which are – (1) Farm profile; (2) Infrastructure and access; (3) Technology and innovation attributes. The structural attributes segment comprises of four main determinants, which are (1) Social structure; (2) Resource needs and support; (3) Institutional factor; (4) Association and organisation.

Table 3 shows main determinants for each segment and the frequency of the determinants being mentioned in the reviewed articles.

**TABLE 3:** Segment, main determinant and the frequency mentioned in the study.

Segments	Main determinant	Frequency
Farmer’s individual attributes	Education and knowledge	201
	Motivation and participation	171
	Gender and demographic	107
Ecosystem and innovation attributes	Farm profile	109
	Infrastructure and access	141
	Technology and innovation attributes	63
Information channel attributes	Extension and training	196
	Communication and information	57
Structural attributes	Social structure	135
	Resource needs and support	193
	Institutional factors	26
	Association and organization	64

### Farmers’ individual attributes

This first segment comprises of main determinants and associate determinants identified in the reviewed articles related to the determinants associated with the farmers individually. The first main determinant is “education and knowledge”. The determinant comprises of several associate determinants such as farmer’s background of education which is mentioned 94 times. The other associate determinants are farmers’ awareness (26 times), knowledge (41 times) and experience (40 times). The other main determinant in this segment is “motivation and participation”. The associate determinant is attitude toward innovation (21 times), motivating factors (5 times), perceived financial benefit (25 times), participation in innovation (18 times), perceived benefit (61 times), perception of risk (28 times), self-interest (3 times) and value co-creation (10 times). The final main determinant for this segment is “gender and demographic” which comprises of age (40 times), general gender factor (39 times), gender of household head (7 times), household size and wealth (19 times) and marital status (2 times).

### Ecosystem and innovation attributes

The second segment of this study contains determinants related to the farm, infrastructure and the innovation itself. The first main determinants in this segment are “infrastructure and access”. There are 7 associate determinants which are basic farm infrastructure (14 times), farm irrigation (9 times), market accessibility (25 times), farm and plot location (36 times), farm/plot management and condition (18 times), farm system (11 times) and access to technology/innovation (28 times). Besides that, the other main determinant in this segment is “farm profile”. The associate determinants are farm/plot size (71 times), land ownership status (32 times) and soil type (6 times). The final main determinants are “technology and innovation attributes” which are divided into 8 associate determinants – compatibility of innovation (14 times), complexity of innovation (10 times), ease of use (7 times), innovation attributes (10 times), observability of innovation (2 times), trialability of innovation (6 times), relative advantage (7 times) and perceived control of innovation (7 times).

### Information channel attributes

This segment comprises determinants associated with diffusing information and technique which lead to farmer’s adoption of new technology. The first main determinant in this segment is “extension and trainings”. The associate determinants are extension services (113 times), farmers school (33 times) and training (50 times). The second main determinant is “communication and information” which comprise communication in general (10 times), communication platform/channel (15 times) and information (32 times).

### Structural attributes

The final segment categorized determinants that are related to external factors that are essential and contributed to staple food crop farmers’ adoption of new technology. The first main determinant is “resource need and support”. Associate-determinants under it are cost of innovation (25 times), access to credit facility (45 times), financial capability (29 times), incentive and subsidy for new technology (21 times), availability of labor (61 times) and off-farm income (12 times). The second main determinants are “social structure” which comprises network trust (14 times), social learning (36 times), social network (36 times), social norm (22 times) and social capital (27 times). The third main determinant is “institutional factor”. It consists of need for policy (17 times), need for regulation (7 times) and power structure (2 times). The final main determinant is “association and organization” which the associate determinants are farmers cooperatives (15 times), membership in farmer association/organization (37 times), leadership (5 times) and partnership with other agency (7 times).

**FIGURE 3:**
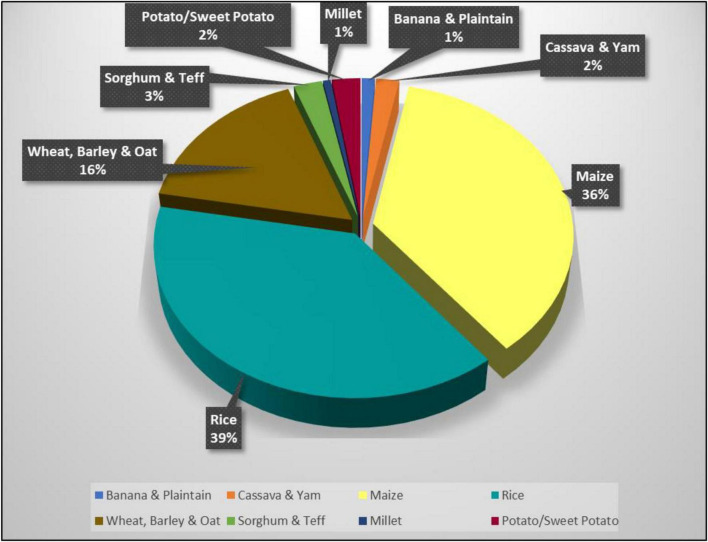
Types of crops identified in the reviewed articles.

This study identified that there are two (2) most frequently reported crops (see Figure 3) which is rice and maize. Drawing on this information, the reported studies and its respected authors based on this categorization is presented in Table 4 for maize and Table 5 for rice.

**TABLE 4:** Attributes, main determinants and list of reported studies – maize.

Attributes	Major determinants	Authors
Farmers’ individual attributes	Education and knowledge	(25–34)
	Motivation and participation	(35–44)
	Gender and demographic	(45–55)
Ecosystem and innovation attributes	Infrastructure and access	(56–61)
	Farm profiles	(62–68)
	Technology and innovation attributes	(69, 70)
Information channel attributes	Communication and information	(71–73)
	Extension and training	(74–84)
Structural attributes	Resource need and support	(85–95)
	Social structure	(9)
	Institutional factor	(96)
	Association and organization	(97–100)

Several studies reported more than one determinant in their findings. The list reported here does not reflects the number of reported determinants in this article.

**TABLE 5:** Attributes, main determinants and list of reported studies – rice.

Attributes	Major determinants	Authors
Farmers’ individual attributes	Education and knowledge	101–106
	Motivation and participation	107–109
	Gender and demographic	110–118
Ecosystem and innovation attributes	Infrastructure and access	119–122
	Farm profiles	105, 123–125
	Technology and innovation attributes	126–131
Information channel attributes	Communication and information	132, 133
	Extension and training	(134–142)
Structural attributes	Resource need and support	101, 103, 143–146
	Social structure	147–155
	Institutional factor	156, 157
	Association and organization	119, 158–162

Several studies reported more than one determinant in their findings. The list reported here does not reflects the number of reported determinants in this article.

### Types of crops, innovations and locations

**FIGURE 4:**
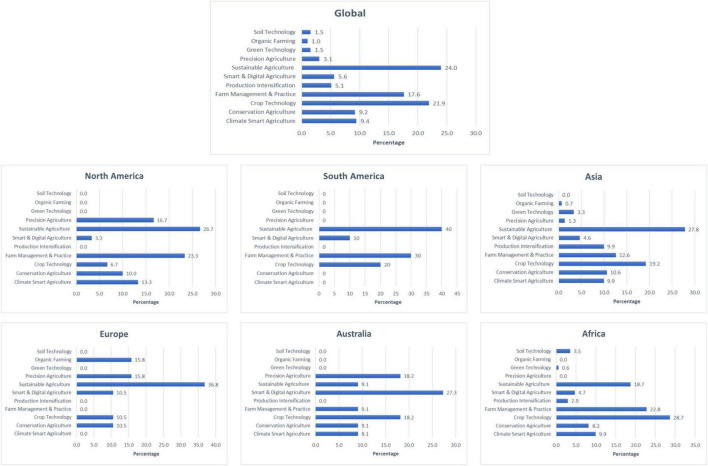
Types of innovation and new technologies according to region.

The findings indicate that there are 11 types of innovation reported in the reviewed articles. Innovations related to sustainable agriculture are the highest (94 articles). It is followed by crop technology (86 articles), farm management and practices (69 articles), climate smart agriculture (37 articles), conservation agriculture (36 articles), smart and digital farming (22 articles), production intensification (20 articles), precision agriculture (12 articles), green technology (6 articles), soil technology (6 articles) and organic farming (4 articles). More than 80% of the reviewed articles reported that their studies were conducted in either Asia or African region. 7.7% of the studies are in North America, 4.8% in Europe, 2.8% in Australia and 2.6% in South America. Figure 4 shows the types of innovations reported according to the region.

The types of crops reported in the reviewed articles are mostly cereal grain where 38.6% of the studies investigated the adoption of innovation amongst rice farmers. The second most reported crops are maize (36.1%) and followed by wheat (16.5%). The remaining 8.8% of the crops reported are sorghum and teff (2.5%), potatoes (2.5%), cassava and yam (2%), plantain (1.1%) and millet 0.7%. Figure 3 shows the proportion of crops reported by articles in this study.

## Discussion

This paper provides a systematic review on the existing literatures in examining determinants of staple food crops farmers’ adoption of new technology or innovation. A rigorous review sourced from two databases has resulted in 392 articles related to determinants of adoption by the staple food crops farmers. The result indicates 4 over-arching segments, 12 major determinants, that comprise of 62 associate determinants. The four segments are farmers, ecosystem, information and structural attributes.

The findings give emphasis on the importance of extension support in promoting adoption of new technology amongst the staple crop farmers. Farmers usually manage their farms based on their personal experience (160, 163, 164). The experience constructs knowledge of farming and is passed from one generation to another. The introduction of new technology or innovation has a learning curve that needs to be achieved by the farmers. Relying on experience alone would not help the farmers to understand or practice any newly introduced innovation. Hence, extension support helps to disseminate information and technique of the new technology or innovation to the farmers. This is in line with other studies that support both extension and education as key factors in determining farmers’ adoption of new technology (102, 136).

Enhancing farmers knowledge about new technologies and innovations hence should be promoted from time to time. It is also reported that farmers involved in trainings have a better understanding of the uptake of crop protection techniques and practices which are monumental to increasing production (165, 166). However, it is important to understand that farmers’ decision to adopt innovation is also influenced by not only the perceived benefits but also risks. Farmers fear that adopting innovations might increase costs, require more labor or could be detrimental to productivity (163, 167).

Farmers are also seen as learning by observing and imitating peers’ action. Social learning hence is one of the factors in determining farmers’ adoption of innovation (129, 168). The working mechanism of social learning might be beneficial considering that small farming is regarded as community work. This environment creates a system itself where farmers will be depending on each other for the source of labor, information, capital and support.

The symbiotic relation between farmer’s element and social elements can also be seen in the concept of the norm. Interestingly, it is identified that both social norm and gender norm are determinants of farmers’ innovation adoption. The prevalence of social norms is reiterated by Fishbein and Ajzen in their Theory of Planned Behavior (169). Several studies adopted and tested this theory where it is reported that farmers adoption of new technology can be explained by the social norm. Farmers do not want to be seen as deviant in their societal norm especially when the technology requires a shift of paradigm in the current method of knowledge. Organic farming and digital farming can be examples of where farmers could have difficulties in leaving their traditional norms. Therefore, it is important for a farming society where norms are paramount to obtain sanction from designated social leaders or institution. If not, Agriculture technology can always be accessible and available to farmers, but the thinking and mindset oriented by culture and religion inversely affect the application [(153), 5].

However, the concept of norms, especially associated with gender, could be affected by the economic practices of various countries. Agriculture and farming activities are usually associated with males dominating the industry. The country’s division of labor could however, shift this perception. For instance, in a study conducted in Vietnam, due to the mass migration of male labor to the city, farming activity in the rural region is largely performed by female farmers. Consequently, the decision to adopt new technology is also influenced by gender norms (170). The monumental role of women as a decision-maker is also echoed in the other study where it is shown that women play a similar role as men when it comes to deciding in adopting new technology (159).

Besides individual and societal role, government and other authoritative agency play a significant role in farmers’ adoption of new technology. Despite low numbers reported in policy and regulation, multiple studies have reported factors such as access to a credit facility, incentive/subsidy, market accessibility as the important one of important determinants. These factors especially related to support and financial assistance are in the realm of authoritative bodies. However, the significant challenges here is identifying and overcoming the void in the roles of governing bodies. Certain government’s policy enables subsidy and incentive for farmers to adopt innovation especially when it is in line with their national agenda. For instance, the Malaysian government spend more than 300 million USD on rice subsidy (171). The role of NGOs in helping to reduce farmers burden in adopting new technology has also been reported in several studies (172–174).

The findings of the review reported in this paper strongly suggest that farmers’ decision to adopt new technology depends on the availability of basic infrastructure within the farm such as irrigation and accessibility to market (110, 117, 175). Having these basic needs, allow farmers to focus on getting improvement in their production. Another focal point that needs to be highlighted is the ability of farm/plot size as a determinant for farmers’ adoption of new technology. It is reported in several studies that farmers with small plot size or small scale farming tend to be more accepting toward new technology or innovation (124, 176, 177). This could be contributed by the fact that farmers with small plots strive to increase their productivity in order to increase revenue. They are unable to enjoy the effect of the “economy of scale” that could be benefitted other large farms.

The analysis of the review also identifies the minimal impact of technology attributes such as complexity, compatibility and ease of use in determining farmers’ adoption of new technology. One of the main reasons could be due to the high reliance on extension services and training. With the help from extension officers, farmers have a higher chance to learn about technological attributes for adoption.

## Conclusion and future studies

Technology adoption at farms producing staple food crops is essential for the food security but also to improve the livelihood of the farmers themselves. This is crucial especially in countries and communities where their socioeconomic largely depends on the local agricultural production. Furthermore, agricultural yields are also essential to ensure a stable household income as well as to achieve daily caloric intake target and balanced nutrition. Understanding the factors contributing to the adoption of new technology or innovation provides opportunities to increase adoption enhance multiple objectives accordingly such as increase productivity, adaptation to climate, sustainable farming and conservation agriculture. The 64 determinants found in this study has been systematically categorized into 12 major determinants and eventually 4 different segments.

This simplification aims to provide both academics and policymakers with the birds-eye view on the current factors that lead to staple food crops farmers adoption of the new technology. The determinants however, also depend on the targeted demographic profiles. A different demographic profile requires a different approach. Future studies hence should examine the process of disseminating new technology, inclusive of participation, information and communications technology-enhanced, and hands-on experience. Future research should also explore fast expanding areas such as digital farming and green technology.

This paper recognizes the importance of examining the determinants for new technology adoption of staple food crop farmers to overcome current global challenges associated with food security especially during the pandemic. This paper presented the outline and summary of past studies related to innovation adoption by farmers of staple food crop for the past 15 years. Based on the systematic review, 12 major determinants of farmers’ adoption have been identified which are education and knowledge, motivation and participation, gender and demographic, farm profile, infrastructure and access, technology and innovation attributes, extension and training, communication and information, social structure, resource needs and support, institutional factors, and association and organization. Types of crops, innovations and locations were also ascertained.

## Data availability statement

The original contributions presented in this study are included in the article/Supplementary material, further inquiries can be directed to the corresponding author.

## Author contributions

AA: conceptualization, writing – original draft, formal analysis, and methodology. SM: supervision, writing – review and editing, and visualization. Both authors contributed to the article and approved the submitted version.
