# Supplementary material for: Getting ahead of the pandemic curve: A systematic review of critical determining factors for innovation adoption in ensuring food security
Source: Front Nutr. 2022 Nov 3;9:986324. doi: 10.3389/fnut.2022.986324 (PMC9669484; doi:10.3389/fnut.2022.986324)
Supplement: Supplementary file 1 [file Data_Sheet_1.PDF]

Supporting Materials 1 – Associate Determinants and Frequency Being Mentioned in the Reviewed Articles

Main Determinants	Associate Determinants	Frequency
Communication & Information	General	10
	Platform/Channel	15
	Information	32
Farm Profiles	Farm Size	71
	Land Ownership	32
	Soil type	6
Infrastructure & Access	Basic Farm Infrastructure	14
	Irrigation	9
	Market	3
	Farm Location	36
	Farm Management and Condition	18
	Farm System	11
	Technology Access	28
	Market Accessibility	22
Education & Knowledge	Education Background	94
	Awareness	26
	Knowledge	41
	Experience	40
Gender & Demographical Factor	Age	40
	Norm	39
	Household head	7
	Household size & wealth	19
	Marital Status	2
Technology and Innovation Attributes	Compatibility of Innovation	14
	Complexity of Innovation	10
	Ease of Use	7
	Innovation Attribute	10
	Observability	2

	Trialability of Innovation	6
	Relative Advantage	7
	Perceived Control of Innovation	7
Social Structure	Network Trust	14
	Social Learning	36
	Social Network	36
	Social Norm	22
	Social Capital	27
Extension & Training	Extension	113
	Farmers School	33
	Training	50
Motivation & Participation	Attitude Towards Innovation	21
	Farmer Motivation	5
	Financial Benefit	25
	Participation in Innovation	18
	Perceived Benefit	61
	Risk Perception	28
	Self Interest	3
	Value Co-Creation	10
Resource Need & Support	Cost of Innovation	25
	Access to Credit Facility	45
	Financial Capability	29
	Incentive & Subsidy	21
	Labour Availability	61
	Off Farm Income	12
Institutional Factor	Policy	17
	Regulation	7
	Power Structure	2
Association & Organization	Farmers Cooperatives	15
	Farmer Association/Organisation	37
	Leadership	5
