# Supplementary material for: Getting ahead of the pandemic curve: A systematic review of critical determining factors for innovation adoption in ensuring food security
Source: Front Nutr. 2022 Nov 3;9:986324. doi: 10.3389/fnut.2022.986324 (PMC9669484; doi:10.3389/fnut.2022.986324)
Supplement: Supplementary file 2 [file Data_Sheet_2.PDF]

Supporting Material 2 – Word String for Database Search

Database	Search String
WOS	TS=(innovation OR technology) AND<br>TS=("diffusion" OR "adoption") AND<br>TS=("food crop" OR "cereal" OR "maize" OR "rice" OR "corn"<br>OR "millet" OR "wheat" OR "barley" OR "paddy") AND TS=(farmer)
SCOPUS	(TITLE-ABS ("innovation") AND ("diffusion" OR "adoption") AND ("food crop" OR "cereal" OR "maize" OR "rice" OR "corn" OR "millet" OR "wheat" OR "barley" OR "paddy")) AND (TITLE-ABS-KEY (farmer))
