# Supplementary material for: Getting ahead of the pandemic curve: A systematic review of critical determining factors for innovation adoption in ensuring food security
Source: Front Nutr. 2022 Nov 3;9:986324. doi: 10.3389/fnut.2022.986324 (PMC9669484; doi:10.3389/fnut.2022.986324)
Supplement: Supplementary file 3 [file Data_Sheet_3.PDF]

Database	Search String	No of Article
WOS	TS=(innovation OR technology) AND<br>TS=("diffusion" OR "adoption") AND<br>TS=("food crop" OR "cereal" OR "maize" OR "rice" OR "corn"<br>OR "millet" OR "wheat" OR "barley" OR "paddy") AND TS=(farmer)	1,278
	TS=(innovation OR technology) AND<br>TS=("diffusion" OR "adoption") AND<br>TS=("food crop" OR "cereal" OR "maize" OR "rice" OR "corn"<br>OR "millet" OR "wheat" OR "barley" OR "paddy") AND TS=(farmer)<br>AND TS= ("survey" OR "interview" OR sample)	448
	TS=(innovation OR technology) AND<br>TS=("diffusion" OR "adoption") AND<br>TS=("food crop" OR "cereal" OR "maize" OR "rice" OR "corn"<br>OR "millet" OR "wheat" OR "barley" OR "paddy") AND TS=(farmer)<br>AND TS= ("survey" OR "interview" OR sample) – Time Span 2006-2021	444
SCOPUS	(TITLE-ABS ("innovation") AND ("diffusion" OR "adoption") AND ("food crop" OR "cereal" OR "maize" OR "rice" OR "corn" OR "millet" OR "wheat" OR "barley" OR "paddy")) AND (TITLE-ABS-KEY (farmer))	1,119
	(TITLE-ABS ("innovation") AND ("diffusion" OR "adoption") AND ("food crop" OR "cereal" OR "maize" OR "rice" OR "corn" OR "millet" OR "wheat" OR "barley" OR "paddy")) AND (TITLE-ABS-KEY (farmer)) AND (TITLE-ABS-KEY (survey OR interview OR sample))	335
	(TITLE-ABS("innovation")AND("diffusion"OR"adoption")AND("food crop"OR"cereal"OR"maize"OR"rice"OR"corn"OR"millet"OR"wheat"OR"barley"OR"paddy")) AND (TITLE-ABS-KEY(farmer)) AND (TITLE-ABS-KEY(survey OR interview OR sample)) AND (EXCLUDE (PUBYEAR,2005) OR EXCLUDE (PUBYEAR,2004) OR EXCLUDE (PUBYEAR,2003) OR EXCLUDE (PUBYEAR,2002) OR EXCLUDE (PUBYEAR,2000) OR EXCLUDE (PUBYEAR,1998) OR EXCLUDE (PUBYEAR,1997) OR EXCLUDE (PUBYEAR,1992) OR EXCLUDE (PUBYEAR,1991) OR EXCLUDE (PUBYEAR,1990) OR EXCLUDE (PUBYEAR,1989) OR EXCLUDE (PUBYEAR,1982) OR EXCLUDE (PUBYEAR,1977) OR EXCLUDE (PUBYEAR,1976)) AND (LIMIT-TO (LANGUAGE,"English"))	296

Combine WOS and Scopus	TS=(innovation OR technology) AND<br>TS=("diffusion" OR "adoption") AND<br>TS=("food crop" OR "cereal" OR "maize" OR "rice" OR "corn"<br>OR "millet" OR "wheat" OR "barley" OR "paddy") AND TS=(farmer)	2,397
	Article excluded – 1 (duplicate value)	130 (261 both scopus and wos)
	Record After Duplicates Removed	2,136
	Record After Screen (Full Text Article Assessed for Eligibility)	740
	Studies Included in Synthesis	392

TS=(innovation OR technology) AND TS=("diffusion" OR "adoption") AND TS=("food crop" OR "cereal" OR "maize" OR "rice" OR "corn" OR "millet" OR "wheat" OR "barley" OR "paddy") AND TS=(farmer) AND TS= ("survey" OR "interview" OR sample)
